# Supplementary material for: Complement C3 Regulates Inflammatory Response and Monocyte/Macrophage Phagocytosis of Streptococcus agalactiae in a Teleost Fish
Source: Int J Mol Sci. 2022 Dec 9;23(24):15586. doi: 10.3390/ijms232415586 (PMC9779060; doi:10.3390/ijms232415586)
Supplement: Supplementary file 1 [file ijms-23-15586-s001.zip › ijms-1878244-supplementary.pdf]

Supplementary Figure

Figure S1.

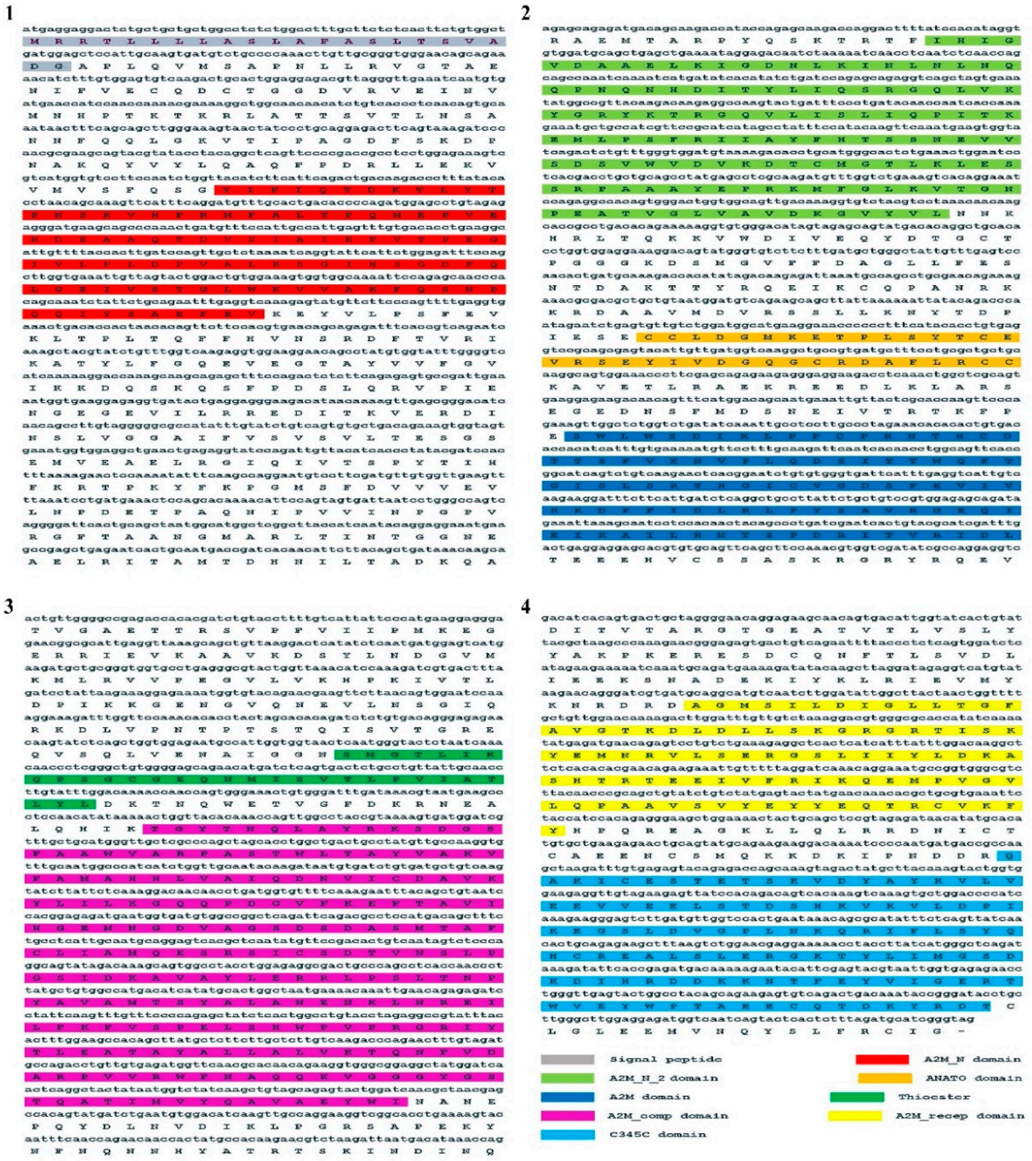

Figure S1. Sequence information of Nile tilapia Component 3 opening reading frame. The Signal peptide is labeled by grey box, the A2M_N domain is labeled by red box, the A2M_N_2 domain is labeled by light green box, the ANATO domain is labeled by orange box, the A2M domain is labeled by blue box, the A2M_comp domain is labeled by purple box, the A2M_recep domain is labeled by yellow box and the C345C domain is labeled by cyan-blue box. Besides, the thioester is labeled by green box.

Figure S2.

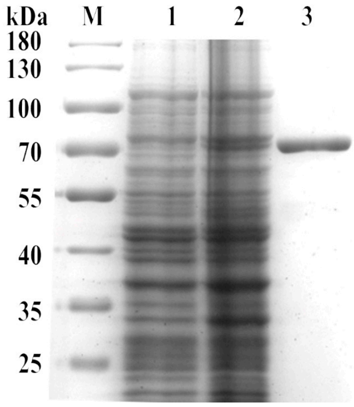

Figure S2. Lane M, markers; Lane 1, the bacteria liquid before IPTG induction; Lane 2, the bacteria liquid was induced with 1 mM IPTG at 37ºC for 6 h; and Lane 3, purified (r)OnC3 fusion protein.
